# Trajectory Clustering-Based Anomaly Detection in Indoor Human Movement

**DOI:** 10.3390/s23063318

**Published:** 2023-03-21

**Authors:** Doi Thi Lan, Seokhoon Yoon

**Affiliations:** Department of Electrical, Electronic and Computer Engineering, University of Ulsan, Ulsan 44610, Republic of Korea

**Keywords:** anomaly detection, indoor human trajectory, DBSCAN, cluster validity index, epsilon parameter, similarity measurement

## Abstract

Human movement anomalies in indoor spaces commonly involve urgent situations, such as security threats, accidents, and fires. This paper proposes a two-phase framework for detecting indoor human trajectory anomalies based on density-based spatial clustering of applications with noise (DBSCAN). The first phase of the framework groups datasets into clusters. In the second phase, the abnormality of a new trajectory is checked. A new metric called the longest common sub-sequence using indoor walking distance and semantic label (LCSS_IS) is proposed to calculate the similarity between trajectories, extending from the longest common sub-sequence (LCSS). Moreover, a DBSCAN cluster validity index (DCVI) is proposed to improve the trajectory clustering performance. The DCVI is used to choose the epsilon parameter for DBSCAN. The proposed method is evaluated using two real trajectory datasets: MIT Badge and sCREEN. The experimental results show that the proposed method effectively detects human trajectory anomalies in indoor spaces. With the MIT Badge dataset, the proposed method achieves 89.03% in terms of F1-score for hypothesized anomalies and above 93% for all synthesized anomalies. In the sCREEN dataset, the proposed method also achieves impressive results in F1-score on synthesized anomalies: 89.92% for rare location visit anomalies (*τ* = 0.5) and 93.63% for other anomalies.

## 1. Introduction

An enormous amount of trajectory data from moving objects is generated due to the development of location-acquisition devices, such as GPS, smartphones, and transportation monitoring systems. The variety and richness of location traces enable a better understanding of the movement behaviors of objects, which provides new applications, including smart transportation, urban development planning, and security surveillance. One of the crucial problems for the above applications is to detect the anomalous trajectories of objects. For taxi services, for example, abnormal trajectories are related to issues, such as traffic congestion, taxi driving fraud, and refusing to take passengers. Therefore, detecting anomalous taxi trajectories may improve the performance of this service [1,2,3,4]. Moreover, anomaly event detection plays a vital role in security surveillance in public spaces. These anomaly events, such as terrorism, violent attack, and fire, can be detected by analyzing the object trajectories in public places [5,6]. Moreover, to guarantee the safety and security of ships during a voyage, their location data are used to detect outlying trajectories and remind other ships to take the necessary avoidance actions [7,8]. Similarly, to ensure aircraft safety, data recorded from current flights are monitored and analyzed constantly. Once anomalous data patterns are detected, they are informed to the flight monitoring system to handle them instantly [9,10,11,12,13]. On the other hand, those studies focused on detecting anomalous trajectories of objects in outdoor spaces.

**Figure 1:**
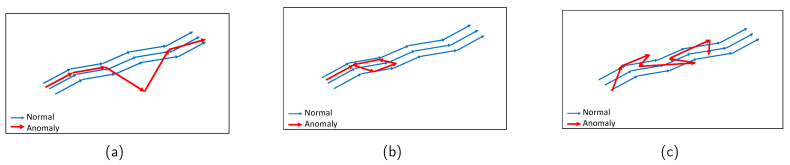
Illustration of anomalous trajectory types: (**a**) Rare location visit anomaly. (**b**) Detour anomaly. (**c**) Random route anomaly.

In recent times, the location data of objects in buildings have been gathered because of the development of indoor navigation systems. This has attracted increasing attention from researchers in new fields. For example, customers’ shopping behavior can be identified by analyzing their historical trajectories in supermarkets. This allows managers to improve product placement and the layout of the supermarkets [14]. Furthermore, human location prediction technologies in indoor spaces have been developed recently and are an important part of location-based services. Predicting where people will be in office buildings can help better understand their intentions and enhance their quality of life [15,16]. Nevertheless, there are few works on discovering anomalies in indoor human movement, which plays an important role in security surveillance and identifying emergencies. Therefore, this study develops a framework to detect anomalies in indoor human trajectories. In particular, two anomaly types are studied, i.e., rare location visit and route anomalies. A rare location is where humans rarely visit or are prohibited from visiting. Therefore, a person’s behavior may be considered abnormal if he/she accesses rare places. In addition, route anomalies can occur when people travel along unusual routes, such as detours or random routes. These anomaly types are illustrated in Figure 1.

There are several challenges in anomaly detection in indoor human movement. First, detecting anomalies in urgent situations has a tight time constraint. This task requires anomaly detection as fast as possible to handle the occurring situations instantly. Hence, existing methods that detect anomalous trajectories with no time limits are unsuitable [3,17,18,19]. Second, the distance between trajectories in indoor spaces is different from outdoor spaces because indoor trajectories are limited by entities, such as rooms, corridors, and stairs. Therefore, anomaly detection methods using existing distance functions, including Euclidean distance, longest common sub-sequence (LCSS) [20], dynamic time warping (DTW) [21], and edit distance on real sequence (EDR) [22], are ineffective for indoor trajectory data. In addition, indoor human movement behavior may be represented by location traces and the semantics of data points. For example, consider the behavior between two people. One stays in a meeting room, the other in a coffee room. Assume that these two rooms are next to each other. Their behavior is entirely different in this case, despite their close proximity. Therefore, semantic information should be considered when estimating the similarity between indoor trajectories. Finally, existing anomaly detection methods do not provide an effective way to choose algorithm parameters, which may degrade the detection performance. For example, the performances of distance-based and density-based methods are affected by the distance and density thresholds [17,23]. Clustering-based anomaly detection methods require input parameters for the clustering algorithm, such as the number of clusters in K-Means, spectral clustering, and hierarchical clustering. DBSCAN requires two input parameters: The minimum number of points to form a new cluster (MinPts) and the radius to find the neighbors (Eps). Choosing an appropriate value of Eps is essential for performing DBSCAN; most studies determined this value manually.

In this work, a DBSCAN-based anomaly detection method is developed, which addresses the above challenges. First, this study detects anomalies in a short time to satisfy time requirements. A time window is chosen to process trajectories. The window size is small enough to meet the time constraint. Next, a novel trajectory similarity metric called the longest common sub-sequence using indoor walking distance and a semantic label (LCSS_IS) is proposed. This metric uses indoor walking distance and semantic labels when calculating the similarity between trajectories. Finally, the proposed method detects human trajectory anomalies based on DBSCAN. The DBSCAN cluster validity index (DCVI) is proposed to determine the Eps value. Three elements were considered when designing this index: The inter-cluster distance, the intra-cluster distance, and the distance between outliers and clusters. DCVI estimates the clustering quality of DBSCAN based on the separation between clusters, the compactness within each cluster, and the separation between outliers and clusters. In summary, the main contributions of this work are presented as follows:

- A novel two-phase anomaly detection framework is proposed to identify anomalous human trajectories in indoor spaces. Instead of estimating a trajectory over a long time, the trajectories in a short duration are considered, and an alarm is given when an anomaly is detected. This ensures timely anomaly detection in urgent situations.
- A new similarity metric LCSS_IS is proposed using indoor walking distance and semantic labels to discover the similarity between trajectories in indoor spaces. In addition, a new cluster validity index for DBSCAN called DCVI is proposed to choose the Eps value. Eps is an important input parameter of DBSCAN, which directly affects the performance of the clustering algorithm.
- The proposed anomaly detection method is evaluated using two real-world datasets. The results show that the proposed method outperforms the other anomalous trajectory detection methods over various anomalous trajectory types.

The paper is structured as follows. Section 2 discusses related works and Section 3 defines the anomaly detection problem. The methodology is described in Section 4. Then, the algorithm performance is evaluated in Section 5. Finally, the conclusion is reported in Section 6.

## 2. Related Works

This section briefly reviews the related works in anomalous trajectory detection. The existing methods are divided into two categories: Methods that do not use similarity metrics and methods that do. The first category discovers the “few and different” characteristics of anomalies to detect them. Zhang et al. [18] introduced the isolation-based anomalous trajectory (iBAT) method to exploit anomalies using the isolation forest (iForest) algorithm [24]. They produced a random tree of trajectories and then adopted the iForest algorithm. The trajectories were split recursively until most were isolated. The trajectories following shorter paths are considered outliers because they were isolated faster than normal trajectories. The authors in [25] designed a technique called maximal anomalous sub-trajectories (MANTRA) to detect temporally anomalous sub-trajectory patterns from an input trajectory. By analyzing the distinctive characteristics of anomalous sub-trajectories, they refined the search space into a disjoint set of anomalous sub-trajectory islands. The resulting set of maximal anomalous sub-trajectories is determined on the anomalous islands.

The second category has attracted more research attention. In particular, there have been various methods using similarity metrics for detecting trajectory anomalies, including the extensible Markov model (EMM)-based, distance-based, density-based, and clustering-based methods. The EMM combines a Markov chain with a clustering algorithm, which detects anomalous trajectories [26]. Each node of EMM is a cluster of location points, which is represented by a cluster model. Depending on the distance between a new point and clusters, the point is grouped either into one of the existing nodes or forms a new node. A point is marked as an anomaly if it belongs to one of two situations: The point forms a new node, or the point belongs to a node whose occurrence probability or whose transition probability is lower than a given threshold. A trajectory is considered anomalous if it contains at least one abnormal point.

The distance-based anomalous trajectory detection method calculates the similarity between trajectories using a distance function. A similarity threshold is given to identify abnormal trajectories [27,28]. Zhu et al. introduced the time-dependent popular routes-based trajectory outlier detection (TPRO) method [27]. In this study, the most popular routes on each timestamp were used to detect the temporal anomalies. The trajectory datasets were divided into groups using a partitioning strategy. A reference trajectory represents each group. The edit distance between the reference trajectory of each group and the popular routes in the given city was then calculated. The anomalous trajectory groups were detected if the distance between its reference trajectory and the popular routes was larger than a given distance threshold. Saleem et al. presented the road segment partitioning towards anomalous trajectory detection (RPAT) algorithm [28]. The trajectories were divided into sub-trajectories based on the road segments. The speed, flow rate, and visited time were features used to calculate the score for each sub-trajectory. The score of each trajectory is the total of its sub-trajectory scores. Trajectory scores above a user-specified threshold are anomalies.

In the density-based anomalous trajectory detection method, the neighbor density of trajectories is estimated to detect anomalies. A previous study [17] proposed the trajectory outlier detection (TRAOD) algorithm by investigating the partition and detection strategy in finding sub-trajectory outliers. A new distance function was proposed, which comprises three components: Perpendicular distance, parallel distance, and angle distance. The density of sub-trajectories was determined using a distance threshold. A sub-trajectory is anomalous if its density is smaller than a threshold. The study in [23] also introduced a distance measure that uses intra-trajectory and inter-trajectory features. The distances between trajectories were first calculated to find the neighbor density of each trajectory. The anomalous trajectories were then detected based on the density threshold.

Clustering-based methods group trajectories into clusters using an appropriate clustering algorithm. Anomalies are detected if they do not belong to any clusters or belong to clusters that only have a few trajectories. Wang et al. [3] proposed an anomalous trajectory detection method using a hierarchical clustering algorithm to derive anomalous trajectories from a taxi GPS dataset. First, the trajectories that travel between the same source and destination were all extracted. The hierarchical clustering algorithm is then adopted to derive the clusters of trajectories using the edit distance. The clusters that have only a trajectory are finally marked as anomalies. Unlike in [3], the authors in [29] developed a two-phase anomalous trajectory detection framework: Online phase and offline phase. The offline phase finds the clusters of trajectories. In this step, the distance between trajectories was calculated using LCSS, and a hierarchical clustering algorithm was also adopted to group trajectories. In the second phase, a trajectory was marked as an anomaly if it did not belong to any clusters.

The challenge of clustering-based anomaly detection methods is determining the input parameters of an algorithm. For example, the hierarchical clustering algorithm requires the number of clusters as an input parameter. Previous studies [30,31] proposed a few indices to find the number of clusters. These indices estimated the compactness of clusters and the separation between clusters for finding the number of clusters in datasets. DBSCAN requires two input parameters: Eps and MinPts; determining the Eps parameter is more difficult for DBSCAN. Many methods have been proposed to choose the Eps value. A combination of the elbow method and the $k^{th}$ nearest neighbor is used to determine the Eps value for DBSCAN [32]. They found *k* nearest neighbors of all data points in the dataset and sorted them in descending order of k−distance. An Eps value was chosen according to the cutoff point of the sorted k−dist graph. On the other hand, the cutoff point cannot always be identified. A new method to choose the Eps value was proposed to overcome the disadvantage of the elbow method [33]. They automatically found the greatest slope change instead of observing the graph. The Eps value was determined according to the point with the greatest slope. The performance of this method still depended on the shape of the k−dist graph. A new approach was also proposed to determine the Eps value using empty circles [34]. They started by finding all empty circles in the dataset. The radius of the circles was sorted in descending order. The elbow value of this sorted radius was chosen as the Eps value. Nevertheless, similar to the reported method [32], it was difficult to determine the appropriate elbow value if it was unclear. In contrast, in our work, the value of Eps is selected based on an evaluation of the clustering quality. Specifically, a novel DBSCAN clustering validity index called DCVI is proposed to choose the Eps value. DCVI measures the compactness of clusters, the separation between clusters, and the separation between clusters and outliers. The value of Eps is determined according to the maximum value of DCVI.

With anomalous trajectory detection methods, which use distance metrics for determining the similarity of the trajectories, choosing an appropriate distance metric plays a vital role. The most straightforward metric, the Euclidean distance, determines the total distance between all pairs of corresponding points on two trajectories. On the other hand, the Euclidean distance requires two trajectories of the same length, and it is sensitive to noises or equipment recording errors. Several distance measurements have been proposed to address these limitations. For example, LCSS can identify similar common sub-sequences between two trajectories of various lengths. This measurement is also noise-resistant because it provides the space and time thresholds to find similar points of two trajectories [20]. DTW has been applied successfully to time series data and trajectories. DTW is based on defining a cost for aligning two data points and finding the minimum cost to align all points between two trajectories [21]. EDR can also remove noise effects by quantizing the distance between a pair of elements to two values, 0 and 1. This measurement does not require the same lengths of two considering trajectories [22]. With indoor spaces, however, the movement path between two points may not be a straight line segment because it is limited by indoor entities [35]. Therefore, the above metrics are relatively ineffective for measuring the similarity of indoor trajectories.

Motivated by this, the authors in [35] proposed an indoor semantic trajectory similarity measure (ISTSM) that was improved from the EDR. ISTSM integrated semantic labels and indoor walking distance to calculate the similarity of indoor semantic trajectories. On the other hand, the indoor walking distance was only considered if the two points were the same semantic. They directly assigned the penalty value of two different semantic points to a maximum value of 1. In other words, they ignored the space aspect between two points if they were different semantic labels. In contrast, this paper proposes a new distance function called LCSS_IS, which is extended from LCSS. The LCSS_IS uses the indoor walking distance in both situations of the same and different semantic labels for estimating the similarity of the indoor trajectories. Furthermore, the trade-off between space and semantic aspects in LCSS_IS is controlled by pre-defined user parameters.

## 3. Problem Definition

This work aims to detect indoor human movement anomalies in emergencies. When humans move, their location data are recorded, and trajectories may be achieved by connecting their location points. A location point *p* is defined as (x,y,t), where (x,y) are the coordinates of *p* at timestamp *t*. A trajectory $T=\{p_{1},p_{2},\ldots p_{n}\}$, which consists of *n* location points, is the collected location data during a time window *W*. Here *n* is the length of trajectory *T*. The trajectories may have different lengths because some data points may be lost during data collection.

**Problem** **1.** *Given a set of historical location data $L=\{L_{1},L_{2},\ldots L_{N}\}$ of N people who participated in collecting data over the same time duration. Assuming that a set of trajectories $D=\{T_{1},T_{2},\ldots T_{M}\}$ is extracted from the location dataset L using the time window W. M is the number of historical trajectories in D. With a new trajectory $T_{new}$ that comes during a time window W, the anomaly detection framework detects whether $T_{new}$ is an anomaly using the historical trajectory set D.*

## 4. Methodology

In this section, a two-phase framework is first proposed to detect human trajectory anomalies using DBSCAN. To improve the accuracy of the similarity measure between trajectories, a new measure called LCSS_IS is then proposed for indoor human trajectories. In LCSS_IS, the indoor walking distance and semantic information are combined to evaluate the similarity between trajectories rather than relying solely on coordinates’ differences between points as in the original LCSS. Unlike ISTSM, which only uses the indoor walking distance for points that have the same semantic label, LCSS_IS uses it in both cases of the same and different semantic labels. Finally, to improve the clustering performance of DBSCAN, a cluster validity index is proposed to determine the Eps value, an important parameter of DBSCAN.

### 4.1. Clustering-Based Anomalous Trajectory Detection Framework

This section introduces an overview of the two-phase framework for detecting human trajectory anomalies. This section also discusses how to cluster trajectories using DBSCAN and detect anomalous trajectories.

**Figure 2:**
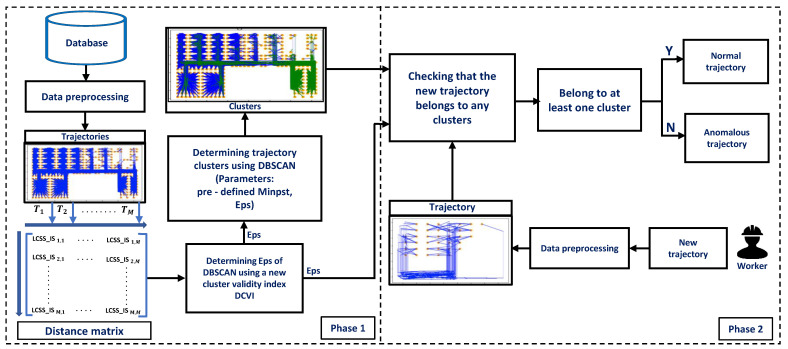
A two-phase framework for detecting human anomalous trajectory.

As shown in Figure 2, phase 1 of the framework groups the trajectories in the database into clusters. First, raw location traces from the database are preprocessed to extract a set of trajectories. Trajectories that have lost many data points from equipment recording errors are removed. Then, a distance matrix of processed trajectories is calculated using a new distance function LCSS_IS that is extended based on LCSS. Finally, we cluster the trajectories using DBSCAN. This step proposes a new cluster validity index DCVI to choose an appropriate Eps value for DBSCAN.

Anomalous trajectory detection is performed in phase 2. A new trajectory is checked to determine whether it belongs to any clusters obtained from the database in phase 1. The Eps-neighbors of the new trajectory in clusters are determined using the Eps value. The new trajectory belongs to the cluster if the number of neighbors is greater than or equal to (MinPts−1). The new trajectory is detected as an anomaly if it does not belong to any clusters. Otherwise, it is a normal trajectory.

### 4.2. Similarity Measure for Indoor Human Trajectories

#### 4.2.1. Longest Common Subsequence (LCSS) Measure

LCSS is a similarity measure for character strings [36]. This measure tries to identify the longest common subsequence between two considering strings. In another study [20], the original LCSS was extended to compare two trajectories of moving objects. Let R and S be two trajectories with length *m* and *n*, respectively, where $R=(\left(r_{x,1},r_{y,1}\right),\left(r_{x,2},r_{y,2}\right),\ldots ,\left(r_{x,m},r_{y,m}\right))$ and $S=(\left(s_{x,1},s_{y,1}\right),\left(s_{x,2},s_{y,2}\right),\ldots ,\left(s_{x,n},s_{y,n}\right))$. For a trajectory R, let Rest(R) be the trajectory $Rest\left(R\right)=(\left(r_{x,1},r_{y,1}\right),\left(r_{x,2},r_{y,2}\right),\ldots ,\left(r_{x,m-1},r_{y,m-1}\right))$. LCSS of *R* and *S* trajectories is defined in the following equation [29].

(1) $$LCSS\left(R,S\right)=\left\{\begin{array}{c} 0,\;\mathrm{if}\;m=0\;\mathrm{or}\;n=0\\ LCSS(Rest(R),Rest(S))+1,\;\mathrm{if}\;|r_{x,m}-s_{x,n}|<\alpha \;\;\mathrm{and}\;|r_{y,m}-s_{y,n}|<\alpha \;\mathrm{and}\;|m-n|\leq \delta \\ max\{LCSS(Rest(R),S),LCSS(R,Rest(S))\},\;\mathrm{otherwise} \end{array}\right.$$

where α and δ are pre-defined parameters that depend on the application and the dataset. α is a distance threshold to determine whether two points are matched. If the difference on the X dimension ($|r_{x,m}-s_{x,n}|$) and the difference on the Y dimension ($|r_{y,m}-s_{y,n}|$) between two points $r_{m}$ and $s_{n}$ are smaller than α, the two points are matched. δ controls how far in time to match one point of R to a point of S.

LCSS can calculate the similarity between two trajectories of varying lengths by finding the matched points between two trajectories. This measurement is also robust to noise by giving the distance and time thresholds to find close points of two trajectories. However, in the LCSS measure, the proximity of two points from two trajectories is determined using the differences on X and Y dimensions. This measure ignores the movement constraints of indoor spaces, which are limited by entities such as rooms, corridors, and stairs. The actual travel distance between two points in indoor space is longer than the distance used in LCSS. LCSS is inappropriate for determining the similarity of indoor trajectories. Moreover, LCSS only uses the space distance to determine the trajectories’ similarity and ignores the semantic information of points. Therefore, this paper proposes a new similarity measure called LCSS_IS, which uses indoor walking distance and semantic labels to determine the similarity of trajectories.

#### 4.2.2. Longest Common Subsequence using Indoor Walking Distance and a Semantic Label (LCSS_IS)

To determine LCSS_IS between two trajectories, a navigation graph is first constructed to calculate the indoor walking distance between two points. The navigation graph represents the topology of a floor plan of an indoor space. In this work, the navigation graph is constructed using a connectivity base graph model [37]. The connectivity base graph is defined by vertices and edges. Entities of the floor plan, such as rooms, stairs, and hallways, are decomposed and represented by vertices. Edges are used to connect vertices in the graph. An edge corresponds to a connection between two partitions in the floor plan. Moreover, to represent the proximity between entities in indoor space, each edge is assigned a value according to the distance of entities. The connectivity base graph model has a low computation complexity while maintaining indoor trajectory modeling efficiency.

**Figure 3:**
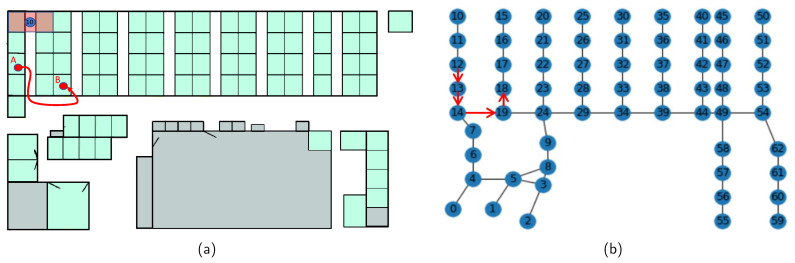
Construction of the indoor navigation graph: (**a**) Floor plan. (**b**) Indoor navigation graph.

In the present work, depending on the floor plans of indoor spaces in datasets, each vertex represents a particular space. For example, in the MIT Badge dataset, a navigation graph is constructed as shown in Figure 3. In this floor plan, the space is divided into small working cubicles for workers. Therefore, each vertex is considered to cover two opposite cubicles and the part of the corridor between these cubicles. Moreover, since the movement characteristics are important for anomaly detection in human movement, vertices are positioned along the corridors. In Figure 3a, vertex 10 covers a space, represented by a small rectangle in the upper left corner of the floor plan. The graph vertices are connected based on the walking routes in the indoor area. To measure the indoor walking distance between the two positions in the floor plan, two vertices in the navigation graph that are the closest to the two positions in the floor plan are determined. The shortest path between the two vertices is defined as the indoor walking distance using the Dijkstra shortest path algorithm [38]. For example, in Figure 3, vertices 12 and 18 are the closest to the A and B points, respectively. Therefore, the distance between points A and B is the shortest path from vertex 12 to 18. This path is marked with red arrows as shown in Figure 3b.

Next, semantic labels need to be assigned to the trajectory points to compute the distance between indoor trajectories using LCSS_IS. Each point is matched with a label using the point coordinates and entity labels in indoor spaces. A list of semantic labels, defined in the MIT Badge and the sCREEN datasets, is shown in Table 1.

A semantic trajectory is then defined as follows:

(2) $$\begin{array}{cc} T= & \left\{\left(\left(x_{1},y_{1}\right),s_{1},t_{1}\right),\left(\left(x_{2},y_{2}\right),s_{2},t_{2}\right),\ldots ,\left(\left(x_{i},y_{i}\right),s_{i},t_{i}\right),\ldots ,\left(\left(x_{n},y_{n}\right),s_{n},t_{n}\right)\right\} \end{array}$$

where $\left(x_{i},y_{i}\right)$ and $s_{i}$ are coordinates and semantic labels of point *i*, respectively, at the timestamp $t_{i}$. *n* is the length of trajectory *T*. LCSS_IS of *R* and *S* trajectories is defined in Equation (3).

(3) $$LCSS\_IS\left(R,S\right)=\left\{\begin{array}{c} 0,\mathrm{if}\;m=0\;\mathrm{or}\;n=0\\ max\{LCSS\_IS(Rest(R),S),LCSS\_IS(R,Rest(S)),LCSS\_IS(Rest(R),Rest(S))+\theta +d\},\\ \mathrm{if}\;IndoorDist(r_{m},s_{n})<\alpha \;\mathrm{and}\;|m-n|\leq \delta \\ max\{LCSS\_IS(Rest(R),S),LCSS\_IS(R,Rest(S)),LCSS\_IS(Rest(R),Rest(S))+\theta \},\mathrm{otherwise} \end{array}\right.$$

where the parameter *d* belongs to the range of (0,1). θ=1−d if two points $r_{m}$ and $s_{n}$ are the same semantic, otherwise θ=0. In this work, *d* represents the spatial proximity between the two points while θ indicates their semantic similarity. When the sum of *d* and θ equals 1, two points are spatially close and have the same semantics. In addition, the value of *d* may be chosen based on the application. For example, d>0.5 if the focus is on the spatial aspect over the semantic information and otherwise. When d=0.5, the spatial proximity and semantic similarity are considered equally. $IndoorDist(r_{m},s_{n})$ is the indoor walking distance between two points $r_{m}$ and $s_{n}$, which is used to measure the spatial proximity between them. Similar to LCSS, α and δ are pre-defined parameters. If α is a distance threshold to determine if two points are close to one another, δ controls how far in time two points are matched.

In addition, two variants of LCSS_IS are also considered: LCSS using indoor walking distance called LCSS_IWD in Equation (4) and LCSS using a semantic label called LCSS_SL in Equation (5).

(4) $$LCSS\_IWD\left(R,S\right)=\left\{\begin{array}{c} 0,\mathrm{if}\;m=0\;\mathrm{or}\;n=0\\ LCSS\_IWD(Rest(R),Rest(S))+1,\mathrm{if}\;IndoorDist(r_{m},s_{n})<\alpha \;\mathrm{and}\;|m-n|\leq \delta \\ max\{LCSS\_IWD(Rest(R),S),LCSS\_IWD(R,Rest(S))\},\mathrm{otherwise} \end{array}\right.$$

(5) $$LCSS\_SL\left(R,S\right)=\left\{\begin{array}{c} 0,\mathrm{if}\;m=0\;\mathrm{or}\;n=0\\ max\{LCSS\_SL(Rest(R),S),LCSS\_SL(R,Rest(S)),LCSS\_SL(Rest(R),Rest(S))+\theta +d\},\\ \mathrm{if}\;|r_{x,m}-s_{x,n}|<\alpha \;\;\mathrm{and}\;|r_{y,m}-s_{y,n}|<\alpha \;\mathrm{and}\;|m-n|\leq \delta \\ max\{LCSS\_SL(Rest(R),S),LCSS\_SL(R,Rest(S)),LCSS\_SL(Rest(R),Rest(S))+\theta \},\mathrm{otherwise} \end{array}\right.$$

Equation (4) shows that LCSS_IWD evaluates the similarity between two points $r_{m}$ and $s_{n}$ only using indoor walking distance. In contrast, the similarity between these two points in the LCSS_SL and LCSS_IS metrics is considered from both spatial and semantic aspects. Nevertheless, to estimate the spatial proximity between two points, LCSS_SL uses their coordinates’ differences between the points as in the original LCSS, while LCSS_IS explores their indoor walking distance. In other words, LCSS_IS is a combination of LCSS_IWD and LCSS_SL.

The above metrics are normalized between 0 and 1, as reported elsewhere [20]. For example, the normalization of LCSS_IS is defined as follows:

(6) $$\begin{array}{c} Norm_{LCSS\_IS(R,S)}=\frac{LCSS\_IS(R,S)}{min(m,n)} \end{array}$$

from $Norm_{LCSS\_IS(R,S)}$, the distance between two trajectories is determined: $Dist_{\left(R,S\right)}=1-Norm_{LCSS\_IS(R,S)}$. When the distance equals 0, two trajectories are the same, while 1 indicates that they are entirely different.

### 4.3. Parameter Determination for DBSCAN

#### 4.3.1. DBSCAN

DBSCAN aims to seek high-density clusters and detect outliers in datasets [32]. The clustering algorithm can also handle datasets with noise and clusters of any shape. In addition, DBSCAN does not require the number of clusters as an input parameter, while some of the clustering algorithms require it [39]. Nevertheless, this algorithm requires two input parameters: Eps and MinPts. The former is the radius to find neighbors, and the latter is the minimum number of points to form a cluster.

Several related definitions of DBSCAN are first stated.

**Definition** **1.** *Point q is an Eps−neighbor of a point p if dist(p,q)≤Eps. dist(p,q) is the distance between two points p and q.*

**Definition** **2.** *Point q is a core point of the cluster if the number of its Eps−neighbors is greater than or equal to MinPts−1.*

**Definition** **3.** *Point q is a border point of the cluster if it is not a core point, but it is an Eps−neighbor of the core point.*

**Definition** **4.** *Outliers are points that are neither core points nor border points of the clusters.*

DBSCAN labels the data points in the dataset as core points if they satisfy Definition 2. Core points are Eps−neighbor to each other belonging to the same cluster. From obtained core points, border points are labeled if they satisfy Definition 3. Outliers are data points that do not belong to any clusters [34].

#### 4.3.2. Determination for Eps Value

In these two parameters of DBSCAN, MinPts can be chosen more easily based on the user’s knowledge of the dataset [32]. In contrast, Eps has a larger effect on the result of clusters and outliers. If Eps is chosen incorrectly, DBSCAN fails to discover clusters of the dataset. For example, when Eps is small, the number of clusters increases, and only a small number of data points are grouped. Moreover, there are many data points found as outliers. When Eps is large, the number of clusters decreases, and there will be few outliers. Therefore, choosing an appropriate Eps value is a challenge for DBSCAN. This study focuses on choosing the value of Eps.

Inspired by existing cluster validity indices [30], this paper proposes a DBSCAN cluster validity index called DCVI. This index is used to choose the Eps value by estimating the quality of DBSCAN. Unlike other clustering algorithms, the clustering results of DBSCAN contain clusters and outliers. Therefore, the quality of DBSCAN is estimated based on measuring the separation between clusters, the separation between outliers and clusters, and the compactness within each cluster. DCVI is defined as the following equation:

(7) $$\begin{array}{c} DCVI=\frac{Min_{l=\{1,\ldots ,\;K-1\},k=\{l+1,\ldots ,\;K\}}InterCD\left(C_{l},C_{k}\right)+Min_{o=\{1,2\ldots ,\;O\},k=\{1,\ldots ,\;K\}}OCD\left(o,C_{k}\right)}{{\left(Min_{k=\{1,\ldots ,\;K\}}IntraCD\left(C_{k}\right)\right)}^{\gamma }} \end{array}$$

In Equation (7), $InterCD(C_{l},C_{k})$ is the distance between clusters $C_{l}$ and $C_{k}$. $OCD(o,C_{k})$ is the distance between outlier *o* and cluster $C_{k}$. $IntraCD(C_{k})$ is the intra-cluster distance of cluster $C_{k}$. γ controls the contribution of the intra-cluster distance to DCVI.

In this work, $InterCD(C_{l},C_{k})$ is the average distance between points in cluster $C_{l}$ and points in cluster $C_{k}$. The minimum distance between outlier *o* and all points in cluster $C_{k}$ is chosen to calculate $OCD(o,C_{k})$. $IntraCD(C_{k})$ is the average distance between points within cluster $C_{k}$. DBSCAN can be considered successful for grouping data points when the outliers are far from clusters, the clusters are separated from each other, and the compactness of clusters is strong. Therefore, the Eps value is chosen when DCVI is maximum.

**Figure 4:**
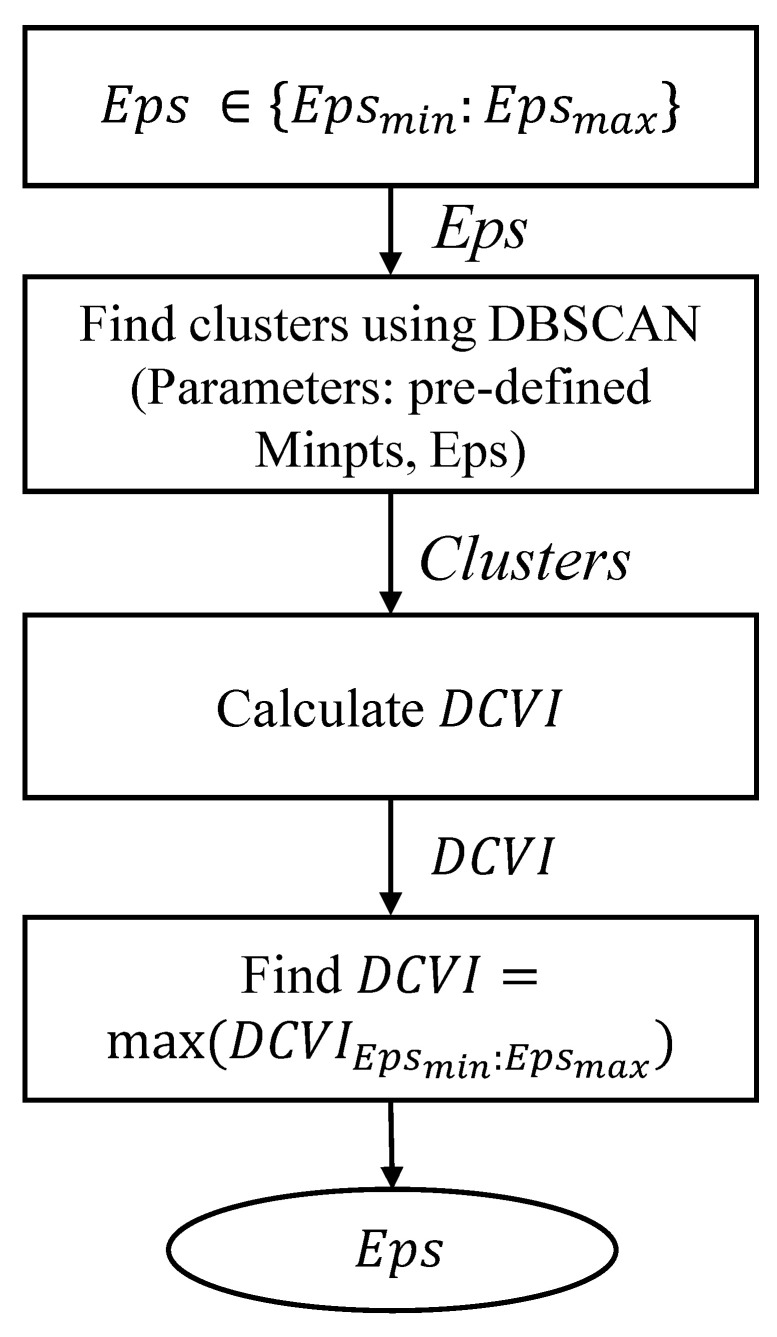
Flowchart for choosing the Eps value using DCVI.

Figure 4 presents a way to determine the Eps value based on DCVI. First, the Eps value is changed from the minimum value to the maximum value of the trajectory distance. According to each Eps value, clusters of the dataset are found using DBSCAN. Then, the value of DCVI is calculated at this Eps value. Finally, the Eps value is chosen according to the maximum value of DCVI.

## 5. Performance Evaluation

### 5.1. Experiment Setup

#### 5.1.1. Datasets and Preprocessing

In this section, two real trajectory datasets are used to evaluate the performance of the proposed method: The MIT Badge dataset and the sCREEN dataset.

- MIT Badge dataset. The MIT Badge (http://realitycommons.media.mit.edu/badgedataset.html, accessed on 8 July 2021) dataset includes the timestamped geographic locations of employees at an IT call center in Chicago from 26 March–17 April 2007 [40]. The location data of workers is estimated by the radio signal strength (RSSI) of the badge assigned to each worker. The in-house positioning system measures the RSSIs of each employee’s badge at various base stations placed around the office. These signals are used to determine the instantaneous position of each badge. Thirty-six workers from three other groups participated in the data collection. The configuration group contains 25 workers. On the other hand, there are only seven in the pricing group and four in the coordinator group. Each recorded data point contains *x* and *y* coordinates at each timestamp. The sampling rate of data is 10 points per minute. As shown in Section 3, each trajectory contains data points collected in a time window *W*. With this dataset, the size of *W* is defined as 10 min. In this case, the maximum trajectory length is 100, and the trajectories shorter than 60 are dropped. Moreover, each day is also divided into timeslots using the time window *W*. Each new trajectory is assigned to a specific timeslot and is detected based only on the history trajectories in this timeslot. The working hours are assumed to be from 9:00 to 18:00, and a day is divided into 54 timeslots. The data are collected within 17 days and divided into two parts for evaluation. The first part accounts for 70% of the total and contains 12 days. This part is used in the first phase of the framework to find the workers’ normal trajectory clusters. The second part is the test dataset, with 30% of the total that contains data within five days. The days for testing are chosen randomly from 17 collected days in the dataset. The experiments are conducted three times with different sets of test days. The output of the algorithm is the average of all run times. The average number of trajectories over timeslots is about 4000 in the first phase. Note that only normal trajectories are used to find clusters in this phase. The average number of trajectories for testing is about 400, with one half for the normal trajectories and the other half for abnormal trajectories.
- SCREEN dataset. The SCREEN (https://vrai.dii.univpm.it/content/screen-dataset, accessed on 1 October 2021) dataset was gathered in a German supermarket during business hours in July 2016 [14]. The location data of customers is collected using sensors installed in shopping carts and baskets. While customers shop for items, sensors send the ultra-wideband signal regularly to anchors placed on the supermarket’s ceiling. The location of the anchors is known. The real-time location system is based on the position of three anchors with the same timestamp to estimate customers’ location. Similar to the MIT Badge dataset, each data point in the sCREEN dataset also contains information about the coordinates and timestamps of each point. Each trajectory is extracted from the location trace using the time window *W* of five minutes. Assuming that the working hours in the supermarket are from 8:00 to 22:00. Unlike workers’ behavior, areas visited by customers in supermarkets do not depend on the time of day, so time is not divided into slots in the sCREEN dataset. All trajectories are grouped to process for detecting anomalies.
  There are 175 carts and baskets to collect data for 29 days. The total number of trajectories is about 40,000. Five days are chosen randomly to process in the first phase and one day for testing in the second phase of the framework. From 6000 trajectories in five chosen days in the first phase, 500 trajectories are randomly chosen for discovering clusters. In addition, 250 trajectories are selected randomly from the chosen day for testing in the second phase. Note that the maximum trajectory length is 436, and trajectories shorter than 200 are removed.

#### 5.1.2. Creating Anomalies for Evaluation

Labeled anomalies are required in the dataset to estimate the algorithm’s performance. Nevertheless, these two datasets do not contain truth anomalies. Therefore, the need to be made for evaluation. There are two usual ways to generate anomalies for datasets in the literature.

First, a hypothesis is given for anomalies based on the difference in behavior between groups in the dataset. The hypothesis is that if one group occurs more frequently than the other, this group is described as a normal group. The remaining group with less occurrence is described as an anomalous group [25,26]. In particular, in the MIT Badge dataset, the configuration group accounts for approximately 70% of the total, while the pricing group accounts for only 20%. Since the movement patterns of two of these groups are different, it is assumed that the movement of employees in the configuration group is normal and in the pricing group is abnormal. The sCREEN dataset does not contain different groups, so there is no hypothesis given for anomalies in this dataset. In other words, the first way to generate anomalies is not used in the sCREEN dataset.

The second way generates anomalies and injects them into datasets [23,41,42]. This study introduces two anomaly types, i.e., rare location visits and route anomalies. Note that generating anomalies in a second way is used for the MIT Badge and the sCREEN datasets.

**Figure 5:**
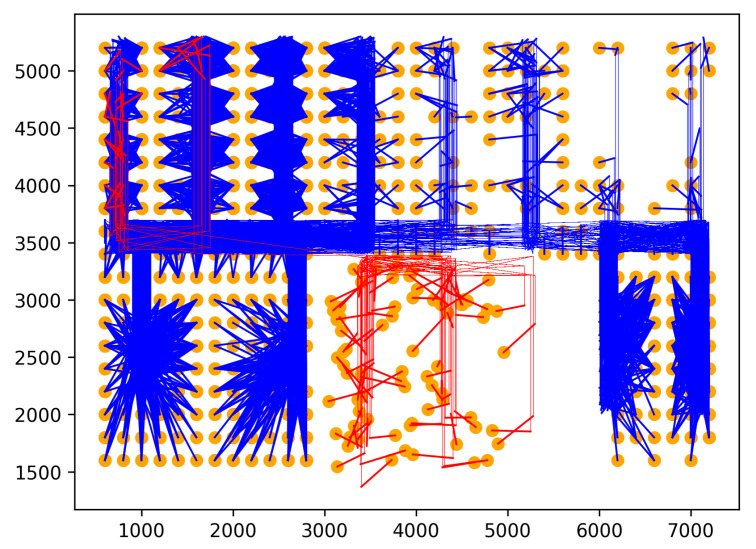
Rare location visit anomaly.

**Figure 6:**
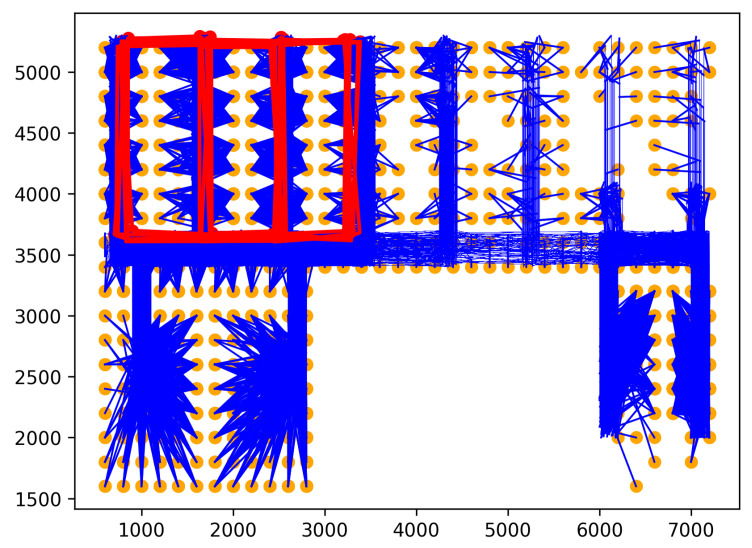
Detour anomaly.

**Figure 7:**
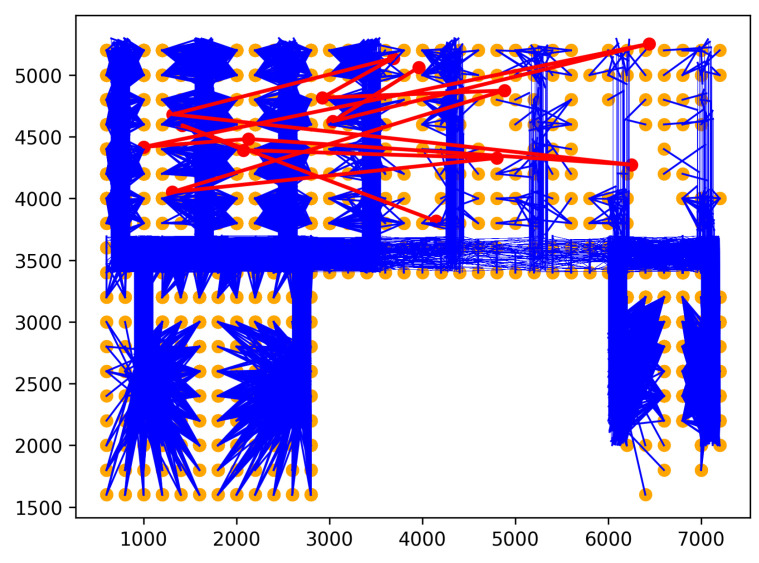
Random route anomaly.

- Rare location visit anomaly. In indoor spaces, the rare location refers to where humans have rarely visited or been prohibited from visiting. For example, in a factory, the prohibited places may be security control and engine rooms that workers can not enter. Moreover, the rare locations can also be places that workers visit only at a specific time. For example, the cafeteria can be a rare location with workers during working hours, even though they may come there for lunch. Similarly, customers in supermarkets or stores are also not permitted to access some locations, including security, staff areas, and warehouses. Therefore, if one person moves to rare locations, his/her behavior may be anomalous. Rare locations need to be identified based on the floor plan and historical trajectories of the dataset to generate this anomaly. First, the floor plan is divided into grid cells. Then, the probability of each trajectory visiting cells is calculated using the history trajectories. The cells with visiting probability are 0 chosen as rare locations. Rare location visit anomalies are generated from the normal trajectories by shifting some points of original trajectories to the rare locations. The number of shifted points is controlled by the parameter τ. For example, τ=0.5 means 50% of the normal trajectory is moved to rare locations. The starting point for shifting is randomly chosen from the original trajectory. As shown in Figure 5, the red trajectory is a generated anomaly, while the blue trajectories are normal.
- Route Anomalies. In some cases, people may travel along unusual routes. For example, when a person tracks an object, he/she often wanders around the object many times. Therefore, he/she may make a detour. In addition, in indoor spaces, incidents, such as fire, may occur suddenly. In this case, people can take random routes to escape. From these aspects, two types of route anomalies are considered: Detour anomaly and random route anomaly. The detour often encircles a specific area. Figure 6 depicts a red detour anomaly. Moreover, a random route anomaly is a list of points, which are selected randomly in the indoor space using a uniform distribution. An example of this anomaly type is shown as the red trajectory in Figure 7.

### 5.2. Parameter Determination for Distance Metric and DBSCAN

#### 5.2.1. Parameter Determination for Distance Metric

This study proposes the LCSS_IS measurement presented in Section 4.2 to measure the distance between trajectories. In this metric, the parameter *d* is set to d=0.5. The value of parameter θ is θ=1−d=0.5 when two points are the same semantic labels. This implies that the weights assigned to spatial closeness and semantic similarity are equivalent when estimating the similarity of the points. Moreover, parameter α=3500 with the MIT Badge dataset and α=5 with the sCREEN dataset. In this work, the value of α is set based on the floor plan scale and the knowledge about human movement behavior in indoor spaces. Here, α is a distance threshold for determining the spatial closeness between points. The parameter δ shows how far in time a point from one trajectory is matched to a point in another trajectory. In this study, the value of δ is set to the maximum point number of two considering trajectories. This means there is no time constraint when estimating the similarity between two points of two trajectories. Since the trajectories are extracted in a short time window, each point from one trajectory is matched to all points in another trajectory over the whole time window.

#### 5.2.2. Determining the Eps Value for DBSCAN

DBSCAN requires two input parameters: MinPts and Eps. Based on previous studies [43,44], MinPts was chosen to be twice the dimensions of data points in the feature space, i.e., MinPts=2×dim. In this work, with the Euclidean, EDR, LCSS, and LCSS_IWD measures, the feature space dimensions for the location points include the x-coordinate, y-coordinate, and timestamp. In contrast, the data space has four dimensions with LCSS_SL, LCSS_IS, and ISTSM because the semantic attribute of the location point is added. Therefore, the MinPts is set to six for the distance metrics in the three-dimensional space and eight for the others in the four-dimensional space.

The value of Eps is chosen using the DCVI metric in Equation (7). Note that the γ parameter, which controls the role of intra-cluster distance, needs to be determined before calculating DCVI. In this work, γ is chosen using the performance of the algorithm when detecting noise. Noise is first generated with a completely different distribution with anomaly types. This step ensures that the determination of the γ parameter is independent of the algorithm performance evaluation when detecting anomalies. Noise is then injected into the dataset as anomalies. The algorithm performance for detecting noise is estimated with the different γ values. Finally, the value of γ, which corresponds to the best performance of the algorithm is selected.

**Figure 8:**
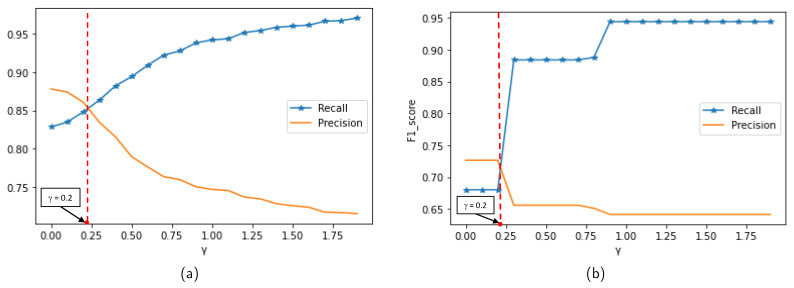
Selecting γ value based on algorithm performance when detecting noise: (**a**) MIT Badge dataset. (**b**) sCREEN dataset.

**Figure 9:**
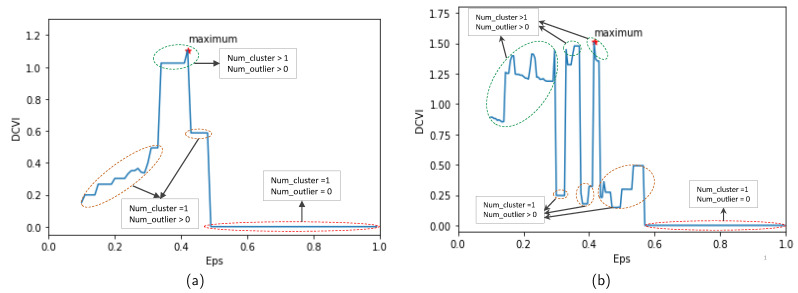
Selecting the Eps value: (**a**) MIT Badge dataset. (**b**) sCREEN dataset.

- Noise creation. Noise points are added to the original trajectory as the following equation [20]:
  (8) $$\begin{array}{c} \left(x_{noise},y_{noise}\right)=\left(x+randn\times X,y+randn\times Y\right) \end{array}$$
  where (x,y) and $\left(x_{noise},y_{noise}\right)$ are coordinates of points before and after adding noise, respectively. randn is a random value created from Gaussian distribution with mean 0 and variance 1. *X* and *Y* are constants that control the proximity between the noise point and the original point. X=7000 and Y=5000 with the MIT Badge dataset and X=10 and Y=10 with the sCREEN dataset. In addition, the ratio of noise point number in each trajectory is chosen randomly in the range [0.3,1]. The starting point for adding noise is chosen randomly from the original trajectory.
- Choosing γ parameter. The value of γ is selected based on the trade-off between the algorithm’s recall and precision. In this work, γ corresponds to the intersection point of recall and precision. This means that the algorithm needs to ensure anomaly detection ability while maintaining precision. Figure 8a,b show the performance of the algorithm when detecting noise for the MIT Badge and the sCREEN datasets, respectively. The value of γ, which equals 0.2 for both datasets, is determined from these figures.
- Choosing Eps parameter. After selecting γ, the Eps value is determined using the DCVI metric, as shown in Figure 4. The Eps value is chosen according to the maximum value of DCVI. Note that, in the MIT Badge dataset, data are divided into timeslots, and the algorithm is performed following each timeslot. Therefore, there are 54 Eps values according to 54 timeslots. Here, this paper shows only one chosen Eps value according to slot 0, which is 0.42 at the red point in Figure 9a. On the other hand, with the sCREEN dataset, data are not divided into timeslots, so only one Eps value (i.e., 0.41 at the red point as in Figure 9b) is selected. In Equation (7), it should be noted that, if the number of clusters and outliers are equal to 1 and 0, respectively, $InterCD(C_{l},C_{k})$ and $OCD(o,C_{k})$ are equal to 0. As in Figure 9, if Eps is too large, there is no outlier, and all trajectories are grouped into one cluster. In this case, DCVI equals the minimum value at 0 according to the part shown by the red ellipse. In addition, if the number of clusters is higher than one and the number of outliers is greater than 0, DCVI is large according to the portion indicated in the green ellipses. The yellow ellipses show that the DCVI is small at Eps values, where only one cluster is found, and the number of outliers is greater than 0.

### 5.3. Result Analysis

In this work, we use three metrics: recall, precision, and F1-score to estimate the algorithm performance. The proposed method is evaluated and compared with four baselines.

- EMM. The work in [26] detects anomalous trajectories using an EMM.
- Density method. This method detects a trajectory as an anomaly if its density is smaller than a given threshold. The density method has been used in studies [17,23,45].
- Hierarchical clustering. The hierarchical clustering-based anomaly detection method is proposed in [29]. This work detects abnormal trajectories based on their closeness with extracted clusters from datasets.
- Spectral clustering. From a previous study [29], the hierarchical clustering algorithm is replaced with the spectral clustering algorithm for finding clusters from datasets. Anomalies are found in the same way in [29].

The baselines, except for EMM, also require a distance measure to calculate similarity in the trajectories. In this work, these baselines use four existing distance measures: Euclidean, EDR, LCSS, and ISTSM. Our DBSCAN-based anomaly detection method is evaluated using seven existing and proposed distance metrics: Euclidean, EDR, LCSS, ISTSM, LCSS_IWD, LCSS_SL, and LCSS_IS. The following subsections present the outcomes of methods over the various anomaly types.

#### 5.3.1. Anomaly as Pricing Group

This subsection only estimates the performance of the methods using the original MIT Badge dataset. Because there is no labeled anomaly in the dataset, a hypothesis is given for creating anomalies. In particular, in the MIT Badge dataset, the configuration group accounts for approximately 70% of the total while the pricing group is only 20%. Therefore, it is assumed that the workers’ movement in the former is normal, while the workers’ movement in the latter is abnormal. Moreover, the number of normal and abnormal trajectories for evaluating the algorithm is chosen equally. The algorithm is fairly estimated owing to the balance between abnormal and normal sample numbers in the test dataset. Table 2 lists the results of baselines and the proposed method for detecting anomalies as the pricing group. In particular, EMM achieves a high recall value of 89.55% compared with a precision of only 66.87%. Because EMM evaluates the abnormality at the level of the trajectory point, and a trajectory is detected as an anomaly if it contains at least one anomalous point. This means that EMM prioritizes seeking the abnormality of the trajectory point. Therefore, there are many normal trajectories detected as anomalies, and EMM precision is low.

The methods, which use distance metrics, evaluate the abnormality at the trajectory level. With these methods, LCSS obtains a higher precision than EDR and ISTSM. One possible explanation is that LCSS aims to find the longest common subsequence between two trajectories, ignoring the unmatched points. Therefore, LCSS tends to seek out the normality of a trajectory rather than its abnormality. This explains why the methods obtain better results in precision. Moreover, in four distance metrics (Euclidean, EDR, LCSS, and ISTSM), the biggest disadvantage of Euclidean, compared with the remaining metrics, is that it can not be applied directly to the trajectories with different lengths. In this work, to use the Euclidean metric, the missed points are interpolated based on existing points. The linear interpolation method was used [46]. After interpolation, the Euclidean distance may estimate the similarity of trajectories quite well because this metric uses the absolute difference between points. Therefore, the performance of methods using Euclidean is comparable to EDR, LCSS, and ISTSM.

ISTSM, which uses space and semantic aspects to determine the trajectories’ similarity, obtains a higher recall than precision for all methods. One possible explanation is that ISTSM prioritizes seeking the difference between trajectories. In ISTSM, if two points have different semantic labels, evenly close in space, a maximum value of 1 is assigned to the substitution cost of the two points. Therefore, trajectories may be detected as anomalies more easily, and recall is high. However, precision is low, so F1-score for this measure is low.

The proposed method with LCSS_IS outperforms the other methods in the F1-score. LCSS_IS, which is extended from LCSS, shows an improved ability to distinguish between two trajectories using the indoor walking distance and semantic labels to determine points’ similarity. This means that LCSS_IS uses the spatial proximity and semantic information to estimate the distance of the trajectories rather than that solely based on the space aspect as LCSS, EDR, and Euclidean. Unlike ISTSM, which only uses the indoor walking distance for the same labels and ignores it for the different labels, LCSS_IS uses the indoor walking distance for both the same and different labels. Moreover, the performance of the two variants, LCSS_IWD and LCSS_SL, are also evaluated. In LCSS_IWD, the indoor walking distance is used to determine the similarity between the two points. Meanwhile, LCSS_SL uses both the space and semantic aspects to estimate the similarity. Note that, in LCSS_SL, the spatial proximity is determined using the coordinates’ norm between two points as in LCSS. As can be seen in Table 2, LCSS_IWD achieves high precision, while LCSS_SL improves recall. Since LCSS_IS is a combination of LCSS_IWD and LCSS_SL, the performance of the proposed method with LCSS_IS is improved in the recall, precision and F1-score.

#### 5.3.2. Synthetic Anomalies

This subsection estimates the performance of the methods for two synthetic anomaly types: Rare location visits and route anomalies. To estimate the anomaly detection ability of the methods over various anomaly types, in the test dataset, abnormal trajectories are replaced according to their type, and the normal trajectories remain.

Table 3 and Table 4 depict the results of detecting rare location visit anomalies on the MIT Badge and the sCREEN datasets, respectively. In the experiments, τ={0.5,1}, which is the ratio of the shifted point number to rare locations in each trajectory. The outcome of methods is better when τ increases. This is because the abnormality is higher when a trajectory remains at a rare location over a longer time. Table 5 and Table 6 show the performance of methods in both datasets when detecting route anomalies.

In the evaluated baselines, EMM achieves the highest recall value with synthetic anomaly types on both datasets. As previously stated, a trajectory is detected as an anomaly by EMM if the trajectory has at least one abnormal point. Moreover, synthetic anomalies contain points with high abnormality, and the points are identified easily by EMM. Therefore, EMM may detect all synthetic anomalies. However, because a trajectory is detected easily as an anomaly by EMM, the precision of this method is low. Hence the F1-score is also low.

The baselines, which use distance metrics, also obtain a very high recall value with all types of synthetic anomalies on both datasets. Because the synthetic anomalies have a strong abnormality at the trajectory level compared to the normal trajectories, they may be detected by the baseline methods. Nevertheless, the precision of the baselines is low. As the baselines detect anomalies using thresholds (i.e., the density threshold in the density method and the distance threshold between a trajectory and clusters in hierarchical and spectral clustering), their performance is affected by the value of the thresholds. However, choosing an appropriate threshold value for detecting anomalies is a challenge. With baselines, thresholds are chosen based on the knowledge about datasets. Therefore, the methods may not obtain the best performance in precision.

In contrast, the proposed method improves precision value compared to the baselines while achieving a high recall value. This is because our method detects anomalies using the Eps value. The appropriate value of Eps is determined based on estimating the clustering quality of DBSCAN using the DCVI metric. Therefore, the performance of the proposed method is improved significantly compared with the baselines in precision and F1-score with synthetic anomalies. Moreover, in the proposed method, LCSS_IS outperforms other distance metrics in the F1-score, except for detecting route anomalies on the MIT Badge dataset. In this case, the F1-score obtains the highest value of approximately 96% with the variant

LCSS_IWD. With LCSS_IS, this method also achieves a high F1-score of approximately 95% and outperforms the other baselines.

Because normal trajectories are maintained in the test dataset when estimating the method performance over other anomaly types, the result of FalsePositive samples does not change for each method. Therefore, if the result of TruePositive samples of a method is the same as with different anomaly types, the F1-score of the method is equal according to these anomaly types. For example, the F1-score for detecting detour and random route anomalies is the same following each method in the sCREEN dataset.

#### 5.3.3. Effect of the MinPts Parameter on Performance

**Figure 10:**
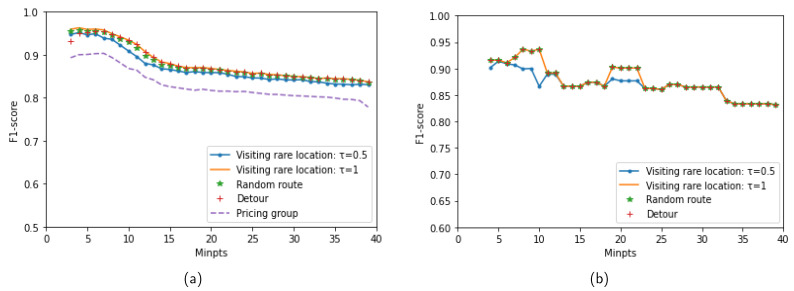
Performance of the proposed method when varying MinPts: (**a**) MIT Badge dataset. (**b**) sCREEN dataset.

This subsection presents the performance of the proposed method over various anomaly types when varying the MinPts parameter of DBSCAN. It is known that MinPts has less influence than Eps on the clustering quality of DBSCAN. The value of MinPts often is chosen based on the user’s knowledge about datasets [45] (i.e., MinPts can be set to twice the dimensions of the data). In this experiment, the MinPts change in the range of [3,40]. With a given value of MinPts, an appropriate value of Eps is determined using the DCVI metric. Then, the chosen Eps value is used for clustering trajectories and detecting anomalies. Figure 10a,b show the results in terms of the F1-score for the MIT Badge and sCREEN datasets, respectively. Note that only the LCSS_IS measure is used in this experiment. The trend of changing performance is similar to all anomaly types. The proposed method achieves the best results around MinPts = 8 on the two datasets. When MinPts>10, the performance of the proposed method is decreased. This may be explained by the fact that many trajectories are detected as noise when the MinPts that are chosen are too large, which may affect the clustering quality of DBSCAN. Therefore, the effectiveness of detecting anomalies is degraded.

#### 5.3.4. Evaluation for Processing Time

This subsection evaluates the processing time for one trajectory in phase 2 of the framework. This is the average processing time of one trajectory in the test dataset. A comparison between the processing time of the proposed method and baselines is shown in Table 7.

The processing time in the sCREEN dataset is longer than the MIT Badge dataset. This is because the average length of one trajectory in the sCREEN dataset is longer than that in the MIT Badge dataset (i.e., 327 in sCREEN and 62 in MIT Badge). The hierarchical and spectral clustering-based methods have lower processing time than other methods. Those two methods only compare a trajectory with the cluster centers. Therefore, the processing time is short. With the DBSCAN-based anomaly detection method, the checked trajectory is compared with trajectories in clusters to decide whether it is an anomaly or normal. The density method processes the checked trajectory based on its density. This method compares the checked trajectory to all trajectories in the dataset to find its neighbors. Therefore, the density method has the longest processing time compared with other methods.

The processing time for other distance measures is also different in the same method. Table 7 shows that Euclidean distance has the lowest processing time (i.e., 0.004 in the MIT Badge dataset and 0.006 in the sCREEN dataset). This can be explained that Euclidean distance is a simple measure with a low computation complexity compared with other measures. The proposed measures and LCSS also have a lower processing time than EDR and ISTSM. For example, LCSS_IS only need 0.405 s for the MIT Badge dataset and 1.01 s for the sCREEN dataset. In urgent situations, this processing time can be accepted to detect anomalies effectively.

## 6. Concluding Remarks

This paper proposed a two-phase framework for detecting indoor human trajectory anomalies based on DBSCAN. This proposed method discovered trajectory clusters in the dataset. A newly coming trajectory is detected as an anomaly if it does not belong to any clusters of trajectories. A novel measure called LCSS_IS was proposed to determine the similarity of the trajectories, which was extended from the original LCSS for discovering the features of indoor human movement. In particular, the indoor walking distance and semantic information were combined to estimate the similarity of trajectories. Therefore, LCSS_IS measured the distance of trajectories in indoor spaces more precisely than existing distance metrics. Furthermore, a novel cluster validity index, DCVI, was proposed to choose the Eps parameter for DBSCAN. DCVI was designed to measure the separation between clusters, the separation between clusters and outliers, and the compactness within each cluster. An appropriate Eps value was determined corresponding to the maximum value of DCVI. The proposed method was evaluated on two real datasets: MIT Badge and sCREEN. The different anomalous trajectory types were also detected in this work. The proposed method showed impressive performance and outperformed the baselines.

In this work, there are a few limitations. First, several features of trajectory data, such as speed and moving direction, were not considered for calculating the distance between trajectories. Second, the proposed method was not evaluated on datasets with complex floor plans, such as buildings with many floors. We plan to extend this work to address the above limitations in a future study.

## Figures and Tables

**Table 1:** List of semantic labels in datasets.

Dataset	MIT Badge			sCREEN		
	Working Space	Rest Space	Travel Space	Category of Products	Entry and Exit	Travel Space
	Configuration	Kitchen	Corridors	Fruit and vegetables	Entrance	Main pathway
	Pricing	Coffee	X	Dairy	Checkout	Racetrack
**Semantic labels**	Coordinator	Centers	X	Frozen	X	X
	Printer, Copy	X	X	Drinks	X	X
	Meeting	X	X	Bakery	X	X
	Base space	X	X	Beauty	X	X

**Table 2:** Results with anomaly as pricing group.

Method	Measure	Recall	Precision	F1-score
EMM		0.8955	0.6687	0.7635
Density	Euclidean	0.7634	0.7241	0.7408
	EDR	0.9929	0.6394	0.7779
	LCSS	0.8221	0.7692	0.7927
	ISTSM	0.8301	0.7005	0.7598
Hierarchical Clustering	Euclidean	0.8702	0.7206	0.787
	EDR	0.8923	0.6738	0.7676
	LCSS	0.7826	0.7082	0.7435
	ISTSM	0.9288	0.6701	0.7783
Spectral Clustering	Euclidean	0.8681	0.7198	0.7858
	EDR	0.8823	0.6964	0.7782
	LCSS	0.816	0.7227	0.7664
	ISTSM	0.9207	0.6737	0.7777
Proposed	Euclidean	0.7897	0.8214	0.8052
	EDR	0.9035	0.7479	0.8179
	LCSS	0.8363	0.8085	0.8216
	ISTSM	0.8624	0.7134	0.7805
	LCSS_IWD	0.6856	0.8817	0.771
	LCSS_SL	0.9539	0.8022	0.8708
	LCSS_IS	0.8997	0.8838	**0.8903**

**Table 3:** Results with rare location visiting anomaly on the MIT Badge dataset.

τ (%)		0.5			1		
Method	Measure	Recall	Precision	F1-Score	Recall	Precision	F1-Score
EMM		1	0.6082	0.7563	1	0.6082	0.7563
Density	Euclidean	0.9528	0.7746	0.8537	0.9528	0.7746	0.8537
	EDR	0.9964	0.6826	0.8098	1	0.6834	0.8115
	LCSS	0.9911	0.8334	0.9052	1	0.8346	0.9096
	ISTSM	0.8118	0.7011	0.7524	0.9939	0.7963	0.884
Hierarchical Clustering	Euclidean	0.9379	0.7475	0.8285	1	0.7465	0.8532
	EDR	0.8953	0.6542	0.7554	1	0.6787	0.8084
	LCSS	0.9132	0.7085	0.797	1	0.7265	0.8412
	ISTSM	0.968	0.6401	0.7706	1	0.6894	0.8159
Spectral Clustering	Euclidean	0.9383	0.746	0.8277	1	0.745	0.8522
	EDR	0.8766	0.6993	0.7763	1	0.7255	0.8402
	LCSS	0.8973	0.7368	0.8074	1	0.8365	0.9098
	ISTSM	0.8411	0.6448	0.7299	1	0.6837	0.8119
Proposed	Euclidean	0.9941	0.7908	0.8792	1	0.8351	0.9083
	EDR	0.8401	0.7961	0.8153	1	0.8228	0.9025
	LCSS	1	0.8585	0.923	1	0.8585	0.923
	ISTSM	0.8792	0.7189	0.7886	1	0.8141	0.897
	LCSS_IWD	0.741	0.9072	0.8145	0.983	0.927	0.9543
	LCSS_SL	0.9977	0.8114	0.8944	1	0.8117	0.8956
	LCSS_IS	0.9629	0.91	**0.9356**	1	0.9137	**0.9545**

**Table 4:** Results with rare location visiting anomaly on the sCREEN dataset.

τ (%)		0.5			1		
Method	Measure	Recall	Precision	F1-Score	Recall	Precision	F1-Score
EMM		1	0.5828	0.7364	1	0.5828	0.7364
Density	Euclidean	1	0.672	0.8038	1	0.672	0.8038
	EDR	0.748	0.6608	0.7017	1	0.7225	0.8389
	LCSS	1	0.7102	0.8305	1	0.7102	0.8305
	ISTSM	0.884	0.6637	0.7582	1	0.6906	0.817
Hierarchical Clustering	Euclidean	1	0.5176	0.6821	1	0.5176	0.6821
	EDR	1	0.5435	0.7042	1	0.5435	0.7042
	LCSS	1	0.7102	0.8305	1	0.7102	0.8305
	ISTSM	1	0.6443	0.7837	1	0.6443	0.7837
Spectral Clustering	Euclidean	1	0.5252	0.6887	1	0.5252	0.6887
	EDR	1	0.5495	0.7093	1	0.5495	0.7093
	LCSS	0.968	0.7634	0.8536	1	0.7692	0.8695
	ISTSM	1	0.6631	0.7974	1	0.6631	0.7974
Proposed	Euclidean	1	0.7937	0.885	1	0.7937	0.885
	EDR	1	0.8013	0.8897	1	0.8013	0.8897
	LCSS	0.996	0.8111	0.8941	1	0.8117	0.8961
	ISTSM	1	0.6684	0.8013	1	0.6684	0.8013
	LCSS_IWD	1	0.8065	0.8929	1	0.8065	0.8929
	LCSS_SL	1	0.7553	0.8606	1	0.7553	0.8606
	LCSS_IS	0.928	0.8722	**0.8992**	1	0.8803	**0.9363**

**Table 5:** Results with route anomalies on the MIT Badge dataset.

Route Anomaly		Detour Anomaly			Random Route Anomaly		
Method	Measure	Recall	Precision	F1-Score	Recall	Precision	F1-Score
EMM		1	0.6082	0.7563	1	0.6082	0.7563
Density	Euclidean	0.815	0.8229	0.8184	0.815	0.8229	0.8184
	EDR	1	0.6834	0.8115	1	0.6834	0.8115
	LCSS	0.9974	0.8328	0.9076	0.9937	0.8323	0.9057
	ISTSM	0.9915	0.796	0.8828	0.8763	0.773	0.8167
Hierarchical Clustering	Euclidean	1	0.7465	0.8532	1	0.7465	0.8532
	EDR	0.976	0.6734	0.7968	0.99	0.6765	0.8036
	LCSS	0.9903	0.7272	0.8382	0.9846	0.7261	0.8354
	ISTSM	1	0.6894	0.8159	0.9843	0.6439	0.7784
Spectral Clustering	Euclidean	1	0.745	0.8522	1	0.745	0.8522
	EDR	0.9751	0.6338	0.7682	0.9914	0.7239	0.836
	LCSS	0.9954	0.7555	0.8582	0.9988	0.7562	0.8599
	ISTSM	1	0.6837	0.8119	0.9048	0.6614	0.7639
Proposed	Euclidean	1	0.8351	0.9083	1	0.8351	0.9083
	EDR	0.8862	0.8703	0.8761	0.9224	0.8839	0.9007
	LCSS	1	0.8585	0.923	1	0.8424	0.9135
	ISTSM	1	0.8141	0.897	0.9685	0.7052	0.8147
	LCSS_IWD	0.9983	0.9291	**0.9624**	0.9928	0.9287	**0.9597**
	LCSS_SL	1	0.8117	0.8956	1	0.8114	0.8954
	LCSS_IS	0.994	0.9132	0.9515	0.9885	0.9132	0.9489

**Table 6:** Results with route anomalies on the sCREEN dataset.

Route Anomaly		Detour Anomaly			Random Route Anomaly		
Method	Measure	Recall	Precision	F1-Score	Recall	Precision	F1-Score
EMM		1	0.5828	0.7364	1	0.5828	0.7364
Density	Euclidean	1	0.6964	0.821	1	0.6964	0.821
	EDR	1	0.7225	0.8389	1	0.7225	0.8389
	LCSS	1	0.7102	0.8305	1	0.7102	0.8305
	ISTSM	1	0.6812	0.8104	1	0.6812	0.8104
Hierarchical Clustering	Euclidean	1	0.5176	0.6821	1	0.5176	0.6821
	EDR	1	0.5435	0.7042	1	0.5435	0.7042
	LCSS	1	0.7102	0.8305	1	0.7102	0.8305
	ISTSM	1	0.6443	0.7837	1	0.6443	0.7837
Spectral Clustering	Euclidean	1	0.5252	0.6887	1	0.5252	0.6887
	EDR	1	0.5495	0.7093	1	0.5495	0.7093
	LCSS	1	0.7692	0.8695	1	0.7692	0.8695
	ISTSM	1	0.6631	0.7974	1	0.6631	0.7974
Proposed	Euclidean	1	0.7937	0.885	1	0.7937	0.885
	EDR	1	0.8278	0.9058	1	0.8278	0.9058
	LCSS	1	0.8117	0.8961	1	0.8117	0.8961
	ISTSM	1	0.6684	0.8013	1	0.6684	0.8013
	LCSS_IWD	1	0.8065	0.8929	1	0.8065	0.8929
	LCSS_SL	1	0.7553	0.8606	1	0.7553	0.8606
	LCSS_IS	1	0.8803	**0.9363**	1	0.8803	**0.9363**

**Table 7:** Processing time for one trajectory (Second).

Method	Measure	MIT Badge	sCREEN
EMM		0.005	0.325
Density	Euc	0.087	1.07
	EDR	0.75	53.7
	LCSS	0.294	28.9
	ISTSM	0.726	62.1
Hierarchical Clustering	Euc	**0.004**	0.008
	EDR	0.017	0.235
	LCSS	0.008	0.164
	ISTSM	0.028	0.258
Spectral Clustering	Euc	0.011	**0.006**
	EDR	0.026	0.192
	LCSS	0.017	0.089
	ISTSM	0.022	0.228
Proposed	Euc	0.081	0.146
	EDR	0.541	17.1
	LCSS	0.299	6.32
	ISTSM	0.518	17.89
	LCSS_IWD	0.193	0.55
	LCSS_SL	0.512	0.926
	LCSS_IS	0.405	1.01

## Data Availability

Not applicable.
